# The value of combined PET/MRI, CT and clinical metabolic parameters in differentiating lung adenocarcinoma from squamous cell carcinoma

**DOI:** 10.3389/fonc.2022.991102

**Published:** 2022-08-23

**Authors:** Xin Tang, Jiaojiao Wu, Jiangtao Liang, Changfeng Yuan, Feng Shi, Zhongxiang Ding

**Affiliations:** ^1^ Hangzhou Health Promotion Research Institute, Hangzhou Wuyunshan Hospital, Hangzhou, China; ^2^ Department of Research and Development, Shanghai United Imaging Intelligence Co., Ltd., Shanghai, China; ^3^ Department of Radiology, Hangzhou Panoramic Imaging Center, Hangzhou, China; ^4^ Department of Radiology, Key Laboratory of Clinical Cancer Pharmacology and Toxicology Research of Zhejiang Province, Affiliated Hangzhou First People’s Hospital, Zhejiang University School of Medicine, Hangzhou, China

**Keywords:** clinical metabolic parameters, CT, lung cancer, PET/MRI, radiomics

## Abstract

**Objective:**

This study aimed to study the diagnostic efficacy of positron emission tomography (PET)/magnetic resonance imaging (MRI), computed tomography (CT) and clinical metabolic parameters in predicting the histological classification of lung adenocarcinoma (ADC) and squamous cell carcinoma (SCC).

**Methods:**

PET/MRI, CT and clinical metabolic data of 80 patients with lung ADC or SCC were retrospectively collected. According to the pathological results from surgery or fiberscopy, the patients were diagnosed with lung ADC (47 cases) or SCC (33 cases). All 80 patients were divided into a training group (64 cases), an internal testing group (8 cases) and an external testing group (8 cases) in the ratio of 8:1:1. Nine models were constructed by integrating features from different modalities. The Gaussian classifier was used to differentiate ADC and SCC. The prediction ability was evaluated using the receiver operating characteristic curve. The area under the curve (AUC) of the models was compared using Delong’s test. Based on the best composite model, a nomogram was established and evaluated with a calibration curve, decision curve and clinical impact curve.

**Results:**

The composite model (PET/MRI + CT + Clinical) owned the highest AUC values in the training, internal testing and external testing sets, respectively. In the training set, significant differences in the AUC were found between the composite model and other models except for the PET/MRI + CT model. The calibration curves showed good consistency between the predicted output and actual disease. The decision curve analysis and clinical impact curves demonstrated that the composite model increased the clinical net benefit for predicting lung cancer subtypes.

**Conclusion:**

The composite prediction model of PET/MRI + CT + Clinical better distinguished ADC from SCC pathological subtypes preoperatively and achieved clinical benefits, thus providing an accurate clinical diagnosis.

## Introduction

Lung cancer is the second most prevalent malignancy and the first leading cause of cancer-related death globally (1, 2). Nonsmall cell lung cancer (NSCLC) is the most common disease, mainly including lung adenocarcinoma (ADC, ~50%) and squamous cell carcinoma (SCC, ~40%) (3), with a 5-year survival rate of 10%–20%. The survival rates only changed slightly despite advances in treatment strategies in recent years. Given the patient variability and tumor heterogeneity, personalized treatment is the key to improve survival over the current poor prognosis. However, a requirement for personalized medicine is the validation of biomarkers and early identification of pathological types (4, 5). Bronchial fiberscopy and surgical biopsy are still the gold standards for the pathological diagnosis of lung cancer, but they are invasive and nonreproducible and may be accompanied by potential complications and false-negative results (6, 7). Therefore, a more effective, non-invasive and repeatable method needs to be explored.

At present, low-dose computed tomography (CT) scan is the main screening method for diagnosing lung cancer. According to the National Lung Screening Trial, low-dose chest CT screening could help in the early diagnosis of cancer and reduce mortality, which was confirmed in several independent, international and randomized controlled clinical trials. However, low-dose CT screening increased the number of indeterminate nodules, in which the high false-positive rate would lead to over-diagnosis, bringing challenges to the management of pulmonary nodules (8). Considering the limitation of a single imaging modality, positron emission tomography/CT (PET/CT) and PET/magnetic resonance imaging (MRI) were developed to improve the efficacy of diagnosis and treatment of lung cancer.

Moreover, radiomics has attracted great attention in a variety of classification tasks. The significant advantages of radiomics include quantifying morphological parameters of tumors, revealing the characteristics of tissue heterogeneity, reflecting the subtle differences between different tissues and linking the image features with tumor characteristics, thus providing objective and valuable information and suggestions for diagnosis, treatment and prognosis (9–11). In the last few decades, many prediction models based on a single imaging modality (CT, PET and MRI) have been developed for quantifying the tumor microenvironment or predicting tumor pathological type, survival and prognosis (12–17). However, composite radiomics models based on PET/MRI, CT and clinical metabolic parameters were rarely reported in the literature. Therefore, this study aimed to establish a radiomics prediction model based on PET/MRI, CT and clinical metabolic parameters, and to explore its prediction and clinical value for the pathological subtyping of ADC or SCC.

## Materials and methods

### Participants

#### Patient selection

The study was approved by the local Medical Research Ethics Committee (Medical Ethics Number: 2021-008), and informed consent was obtained from all participants. Eighty patients with ADC or SCC, initially diagnosed by CT and PET/MRI and pathologically confirmed at Hangzhou Panoramic Imaging Center from October 2018 to October 2021, were retrospectively included as the study participants.

The inclusion criteria were as follows: All patients had pathologically confirmed ADC or SCC; All patients had only one primary lung cancer lesion; No chemotherapy, radiotherapy or surgical therapy was performed before the imaging scan; Clear whole-body and chest PET/MRI and CT images were obtained before treatment; The PET/MRI examination was performed 40–60 min after injecting 18F-fluorodeoxyglucose (18F-FDG).

The exclusion criteria were as follows: patients confirmed with other pathological subtypes than ADC and SCC; patients had two or more primary lung cancer lesions; five patients with lung ADC and two patients with SCC were excluded because PET images were too blurry (SUVmin < 1); three patients with lung SCC and two patients with lung ADC were excluded because of MRI contraindications; patients with a history of other thoracic malignancies or other systemic malignancies; and patients who had received any form of treatment (such as radiotherapy, chemotherapy and so on) before PET/MRI and CT examination.

### Image acquisition

The imaging data were collected using GE 256-slice spiral CT (GE, USA) and integrated time-of-flight (TOF) PET/MR (GE SIGNA, WI, USA). The PET/MRI instrument consisted of a PET detector with TOF technology (TOF-PET) and the latest generation of 750W 3.0T magnetic resonance.

Patient preparation: Patients were fasted for more than 6 h, and blood glucose concentrations were controlled below 7.8 mmol/L before injecting ^18^F-FDG. The patient was injected with ^18^F-FDG at a dose of 3.7 Mbq/kg, and first scanned using the chest CT instrument 40 min later, followed by a whole-body PET/MR imaging scan. Written informed consents were obtained from all patients or legal guardians before the examination.

CT and PET/MRI scan: CT was first performed with the patient in a supine position from the apex to the base of the lung. The CT scanning parameters were tube voltage 120 kV, tube current 80–350 mA, slice thickness 5 mm, slice spacing 5 mm, helical spacing 0.992:1, scanning speed 158.75 mm/s, rotation time 0.5 s, matrix 512 × 512, and noise index 12.0. The scanning was completed under breath-hold condition. After scanning, the original imaging was set to be thin-section reconstructed automatically. The slice thickness was 0.625 mm, and the slice spacing was about 0.625 mm. The CT imaging parameter was a thoracic axial image (5 mm; 1.25 mm; 0.625 mm). After attenuation correction, the patient underwent PET/MRI scanning. The patient was placed in the supine position and scanned from the top of the head to the middle of the femur; additional scans were performed on the sole if necessary. Five to six beds were set. The acquisition time for each bed was 6 min. PET data were acquired using the 3D mode, TOF technique and point spread function with 2 iterations of ordered subset expectation maximum (OSEM), 28 subsets and a 5-mm Gaussian post-processing filter reconstructed into a 192 × 192. PET data acquisition was performed during the whole-body MRI examination. PET/MR scans of the region from the apex to the base of the lung were then performed, and axial, coronal, and sagittal images were obtained using a dedicated MRI coil for the thoracic region to obtain PET, MRI and PET/MR fusion maps of the whole body and region. [Please refer to our previous study (15) for detailed PET/MRI scanning methods.]. All data were acquired from the same CT and PET/MR instruments. Chest CT axial images (1.25 mm), MRI axial T2WI images and PET images were selected as imaging feature extraction sequences in this study (15, 17).

### Clinical features acquisition

We measured each metabolic parameter with PET VCAR software in the AW SERVICE 3.2 processing workstation of the GE Company. The image analysis mainly used visual and semi-quantitative analyses. The uptake boundary of the primary tumor was determined by the adaptive threshold method (17), which identified 40% of the maximum standardized uptake value (SUV_max_) within the regions of interest (ROIs) as the tumor boundary. PET/MR data of patients were transmitted from the GE PACS database to the local and opened by the software. PET/MR fusion image and PET transverse, sagittal, and coronal images were displayed in the 4 × 4 windows. ROIs could be found by dragging the crosshair. Subsequently, we set the default WL percent of PET to 40% under the left Preferences window, clicked the Insert key on the keyboard to insert the ROI automatic identification box, placed the ROI in the outline of primary lung cancer, and delineated the tumor boundary through the iterative adaptive algorithm. The size of the identification frame was adjusted in different windows in three directions, and high-uptake areas, such as normal tissues and metastatic lymph nodes, were excluded from the ROI range in combination with MRI structural images. Finally, the software automatically generated nine metabolic parameters: volume, relative deviation (REL), threshold (THR), standard deviation (STD), peak (PK), SUV_max_, minimum standardized uptake value (SUV_min_), mean standardized uptake value (SUV_mean_) and total lesion glycolysis (TLG) of the ROI. We collected the aforementioned nine metabolic parameters, besides age, sex and site of disease as the clinical features for the study.

### Tumor segmentation

The investigators were blinded to all data including the image reports, clinical documents and the histopathology of the tumors. Axial chest CT images (1.25 mm) in the DICOM format, axial T2WI images of chest MRI and PET images were imported into the ITK-SNAP software (http://www.itksnap.org) and segmented by two chest radiologists with the experience of 10 and 15 years, respectively. ROI of the primary tumor was segmented layer by layer, and the original image and the corresponding ROI image were saved as Nifti files (nii). Subsequently, the intraclass correlation coefficient (ICC) was used to obtain the inter- and intra-measurer consistency and assess its reliability (18).

### Radiomics analyses

The original images and corresponding ROI images were imported into the uAI Research Portal (https://www.uii-ai.com/en/uai/scientific-research) to perform radiomics analyses, which is a platform containing the package of PyRadiomics (http://pyradiomics.readthedocs.io/en). The workflow of radiomics analyses included feature extraction, feature selection, model construction and comparison and model evaluation.

### Feature extraction

The radiomics features were extracted from the CT, MRI and PET images for modeling. A total of 2264 features were extracted from the ROI of each image from 3 modalities. Specifically, the original image was transformed with 14 image filters to generate additional 24 images. The features for each filter were computed for seven categories, that is, including first-order image intensity statistics (histogram), shape, gray-level co-occurrence matrix (GLCM), gray-level run-length matrix (GLRLM), gray-level size-zone matrix (GLSZM), neighboring gray-tone difference matrix (NGTDM), and gray-level dependence matrix (GLDM).

### Data separation

In the dataset, we set lung ADC as “1” and SCC as “0”, respectively. A total of 80 cases were divided into a training group (64 cases), an internal testing group (8 cases) and an external testing group (8 cases) in the ratio of 8:1:1 to ensure data stability; 3 groups owned similar disease distribution.

### Feature selection

The procedure was performed on the training set. For each single modality, the optimal subset of features was selected from the 2264 features using z-score normalization and operators; the *F* test owned the *P* value of 0.01, and the Least Absolute Shrinkage and Selection Operator (LASSO) set the *α* value of 0.1, thus optimal features avoiding collinearity and overfitting were obtained. Considering Harrell’s guidelines, that dimension of selected features should be less than 10% of the total sample, we selected 5 features in a single imaging modality. At the same time, 12 clinical features were also imported, and the features most correlated with the pathological subtypes of ADC and SCC were selected using the *F* test with the *P* value of 0.1 and LASSO regression with the *α* value of 0.1. Then, the dual- or multi-modality features were combined with the corresponding imaging modality.

### Model construction

Based on the optimal features, the BOX-COX transformation and machine learning classifier (Gaussian process, GP) were applied to construct the models. A total of nine models were constructed as follows: PET prediction model, CT prediction model, MRI prediction model, Clinical prediction model, PET + CT prediction model, PET/MRI prediction model, PET + Clinical prediction model, PET/MRI + CT prediction model and PET/MRI + CT + Clinical prediction model.

### Model evaluation and comparison

ROC curve and confusion matrix were used to evaluate the ability of models to distinguish the ADC from SCC. The area under the curve (AUC) value, F1 score, sensitivity, specificity and accuracy in the training group, internal testing group and external testing group of different models were also calculated. The difference in the AUC of the models was compared using DeLong’s test, and the best model (PET/MRI + CT + Clinical model) was chosen. In addition, the nomogram of this composite model was established to help predict the risk probability of one of the diseases (ADC). The calibration curves were used to compare the predictive output and the actual disease. The decision curves plotted the net benefit at a range of risk thresholds and assessed the utility of models for decision making. Clinical impact curves were also used to determine whether basing clinical decisions on the prediction model would do better than harm based on the specific high risk threshold (19, 20).

### Statistical analysis

Statistical analyses were performed with IBM SPSS Statistics (version 26.0) and R software (version 4.1.2). The clinical features were summarized, in which the categorical features (disease, sex, site) were represented with the counts and percentages, while the continuous features (age, nine metabolic parameters) were represented with the median (25%, 75% quantile). Significant differences among the training set, internal testing set and external testing set were analyzed using the chi-square test for categorical features and Kruskal–Wallis H test for continuous features. A *P* value < 0.05 indicated a statistically significant difference. The ICC was used to evaluate the inter- and intra-measurer consistency of ROI delineation. *F* test and LASSO regression were used to select features, and a GP classifier was used to construct radiomics models. The predictive performance of each model was visually evaluated using ROC curves and confusion matrixes and quantitatively assessed using AUC, F1-score, accuracy, sensitivity and specificity. DeLong’s test was used to compare the difference in the AUC values of different models. Nomogram was plotted to show the risk probability of one of the diseases. Calibration curves, decision curves and clinical impact curves were generated to evaluate the predictive performance and clinical net benefit of the composite model. We used several tools within the R environment, including “rms”, “regplot”, “VRPM”, “foreign” and “rmda”.

## Results

### Clinical demographics

A total of 80 patients were enrolled in the study, and their CT and PET/MR images were taken. **Table 1** shows the clinical information of patients. All patients were divided into training set (*n* = 64), internal testing set (*n* = 8) and external testing set (*n* = 8), in which the proportion of people with ADC was 57.8%, 62.5% and 62.5%, respectively. The median age of the patients was 67 years; the female patients accounted for 25.0%. We classified all cases according to the position of the primary lung cancer and customized “central lung cancer” to “0” and “peripheral lung cancer” to “1”. Other nine metabolic parameters, including TLG, volume, PK, THR, SUV_min_, SUV_max_, SUV_mean_, REL and STD, are also shown in **Table 1**. No significant difference was found among the three subsets for all characteristics (*P* > 0.05).

**Table 1:** Clinical characteristics in the study sample (*n* = 80; current thresh: 42).

Characteristics	All samples (*n* = 80)	Training (*n* = 64)	Internal testing (*n* = 8)	External testing (*n* = 8)	*P *
Disease (ADC)	47 (58.8%)	37 (57.8%)	5 (62.5%)	5 (62.5%)	0.943
Sex (female)	20 (25.0%)	14 (21.9%)	4 (50.0%)	2 (25.0%)	0.266
Position (1)	41 (51.2%)	33 (51.6%)	4 (50.0%)	4 (50.0%)	0.994
Age (year)	67.0 (62.3–72.8)	67.0 (63.0–71.0)	65.5 (60.5–75.0)	67.5 (55.3–74.5)	0.996
TLG (g/mL × cm^3^)	91.5 (37.9–236.4)	81.5 (36.4–228.4)	271.4 (128.9–458.5)	83.5 (40.5–145.1)	0.112
Volume (cm^3^)	13.7 (6.9–33.5)	13.2 (6.6–30.1)	31.1 (13.8–56.5)	12.0 (8.9–30.4)	0.264
Peak	9.4 (5.3–11.9)	9.2 (5.3–11.9)	11.5 (9.0–16.0)	8.9 (4.0–10.3)	0.261
Threshold (/42%)	5.0 (3.2– 6.1)	5.0 (3.2–6.1)	5.9 (4.4–8.3)	4.5 (2.5–5.2)	0.286
SUV_min_	5.1 (3.2–6.2)	5.0 (3.2–6.1)	5.9 (4.4–8.3)	4.5 (2.5–5.2)	0.268
SUV_max_	12.0 (7.7–14.6)	11.8 (7.7–14.5)	14.0 (10.5–19.8)	10.7 (6.0–12.3)	0.285
SUV_mean_	7.0 (4.5–8.8)	6.9 (4.5–8.8)	8.1 (6.5–12.5)	6.4 (3.1–7.6)	0.231
Relative deviation	0.21 (0.20–0.23)	0.21 (0.19–0.23)	0.21 (0.19–0.22)	0.22 (0.20–0.23)	0.716
STD	1.4 (0.9–1.9)	1.4 (0.9–1.9)	1.6 (1.4–2.5)	1.4 (0.5–1.7)	0.298

### Tumor segmentation and ICC evaluation

**Figure 1:**
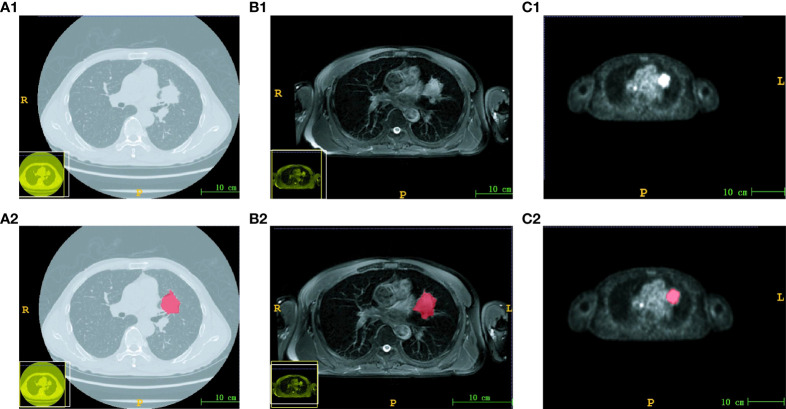
Images and corresponding ROI of three modalities. The original image of CT **(A1)**, MRI **(B1)** and PET **(C1)**. The ROI image of CT **(A2)**, MRI **(B2)** and PET **(C2)**. The scale bar was 10 cm.

The ROI of each image in CT and PET/MR modalities was independently delineated by two experienced radiologists. The original image and the corresponding ROI image are shown in **Figure 1**. Subsequently, the reliability of ROI delineation was evaluated. The results showed that the ICC value of inter- and intra-measurer consistency assessment was 0.943–0.967 and 0.957–0.973, respectively, indicating that it had good reliability and repeatability.

### Model construction and comparison

**Figure 2:**
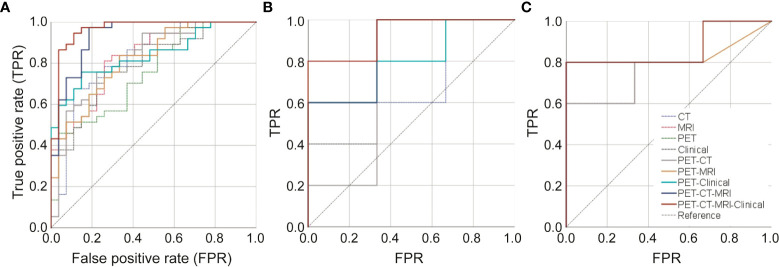
ROC curves of different prediction models. Nine ROC curves in the training set **(A)**, internal testing set **(B)** and external testing set **(C)**.

Based on the delineated ROI, 2264 features were extracted from each image of 3 imaging modalities (PET, MRI and CT). Moreover, 12 clinical features were added to construct a clinical model and a hybrid model. The most relevant imaging features and clinical features were selected using *F* test and LASSO regression sequentially. The detailed information of the selected features is shown in **Figure S1**. The PET prediction model (**Figure S1A**) obtained five most optimal features, including one first-order feature and four texture features, of which glrlm-runlengthnonuniformity and glcm-jointentropy accounted for the top two weight ratios. The CT prediction model (**Figure S1B**) obtained five most optimal features, including one first-order feature and four texture features, of which skewness and glszm-largearealowgraylevelemphasis accounted for the top two weight ratios. The MRI prediction model (**Figure S1C**) obtained five most optimal features, including five texture features, of which glszm-graylevelnonuniformity and gldm-dependencenonuniformitynormalized accounted for the top two weight ratios. The clinical prediction model (**Figure S1D**) obtained three most relevant features, including volume, position and TLG, of which position accounted for the maximum weight ratio. Then, nine models were established by combining features from corresponding modalities, which included PET, CT, MRI, Clinical, PET + CT, PET/MRI, PET + Clinical, PET/MR + CT and PET/MR + CT + Clinical. Therefore, the number of the selected features was 5, 5, 5, 3, 10, 10, 8, 15 and 18, respectively. In the PET/MRI + CT + Clinical prediction model, the mri-GLSZM-GLN weight ratio was about – 0.165, and the pet_shotnoise_GLCM_IDM weight ratio was about 0.145 ( **Formula S2** ). Then, the GP was applied to classify the ADC and SCC. The predictive performances of nine models were characterized using ROC curves (**Figures 2**, **S2**), demonstrating that the PET/MR + CT + Clinical model, named the composite model, had the highest AUC value regardless of the dataset, which was confirmed by **Table 2**. The AUC value of the composite model in the training group, the internal testing group and the external testing group was 0.965 (95% CI: 0.920–1.000), 0.933 (95% CI: 0.746–1.000) and 0.867 (95% CI: 0.593–1.000), respectively. Besides, DeLong’s test was used to compare the AUC of different models. As shown in **Figure S3**, In the training set, significant differences in the AUC were found between the composite model and other models except for the PET/MRI + CT model. At the same time, no significant difference was found between models in the internal testing group or external testing group due to limited data.

**Table 2:** AUC values of different prediction models in the training set, internal testing set and external testing set.

Model	Training	Internal testing	External testing
PET	0.751 (0.632–0.870)	0.733 (0.281–1.000)	0.800 (0.476–1.000)
CT	0.825 (0.717–0.933)	0.733 (0.358–1.000)	0.867 (0.593–1.000)
MRI	0.829 (0.729–0.928)	0.867 (0.593–1.000)	0.800 (0.476–1.000)
Clinical	0.795 (0.686–0.903)	0.800 (0.430–1.000)	0.800 (0.476–1.000)
PET+CT	0.826 (0.721–0.931)	0.733 (0.281–1.000)	0.800 (0.476–1.000)
PET/MRI	0.818 (0.715–0.920)	0.867 (0.584–1.000)	0.833 (0.522–1.000)
PET + Clinical	0.838 (0.742–0.934)	0.800 (0.476–1.000)	0.867 (0.593–1.000)
PET/MRI + CT	0.934 (0.871–0.997)	0.867 (0.584–1.000)	0.867 (0.593–1.000)
PET/MRI + CT + Clinical	0.965 (0.920–1.000)	0.933 (0.746–1.000)	0.867 (0.593–1.000)

AUC was represented with mean and 95% confidence interval (95% CI).

**Figure 3:**
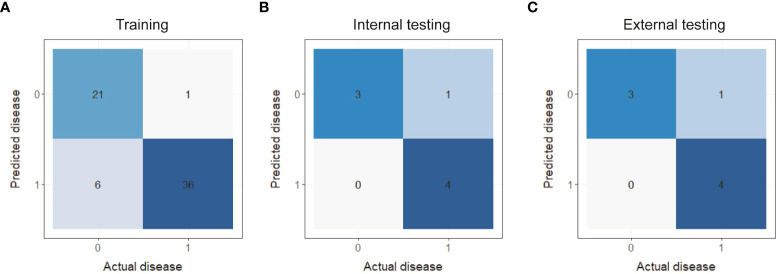
Confusion matrixes of the PET/MRI + CT + Clinical prediction model in the training set **(A)**, internal testing set **(B)** and external testing set **(C)**.

Additionally, the F1-score, sensitivity, specificity and accuracy of each model in the training set, internal testing set and external testing set are listed in **Table 3**. On the whole, the PET/MRI + CT + Clinical model possessed a superior performance in all parameters, especially the accuracy and specificity. Moreover, the confusion matrixes of the composite model are plotted in **Figure 3**. As shown in **Figure 3**, in the training set, 21 of 27 cases of SCC were correctly classified, and only 6 cases were misclassified as ADC, while 36 of 37 cases of ADC were correctly classified and only 1 case was misclassified as SCC. Only one case of ADC was misclassified as SCC and the others were correctly classified in both the internal testing **(** **Figure 3** **)** and external testing sets **(** **Figure 3** **)**. Therefore, it was concluded that the PET/MRI + CT + Clinical model showed good discriminative performance of ADC and SCC.

**Table 3:** F1-score, sensitivity, specificity, and accuracy of different prediction models (IT represented internal testing, and ET represented external testing).

Model	F1-Score			Sensitivity			Specificity			Accuracy			
	Training	IT	ET	Training	IT	ET	Training	IT	ET	Training	IT	ET	
CT	0.795	0.727	0.727	0.838	0.800	0.800	0.630	0.333	0.333	0.750	0.625	0.625	
MRI	0.795	0.727	0.667	0.838	0.800	0.600	0.630	0.333	0.667	0.750	0.625	0.625	
PET	0.756	0.909	0.800	0.838	1.000	0.800	0.481	0.667	0.667	0.688	0.875	0.750	
Clinical	0.747	0.667	0.667	0.757	0.600	0.600	0.630	0.667	0.667	0.703	0.625	0.625	
PET + CT	0.795	0.909	0.800	0.838	1.000	0.800	0.630	0.667	0.667	0.750	0.875	0.750	
PET/MRI	0.795	0.909	0.800	0.838	1.000	0.800	0.630	0.667	0.667	0.750	0.875	0.750	
PET + Clinical	0.769	0.727	0.800	0.811	0.800	0.800	0.593	0.333	0.667	0.719	0.625	0.750	
PET/MRI + CT	0.895	0.909	0.800	0.919	1.000	0.800	0.815	0.667	0.667	0.875	0.875	0.750	
PET/MRI + CT + Clinical	0.911	0.889	0.889	0.973	0.800	0.800	0.778	1.000	1.000	0.891	0.875	0.875	

### Composite model evaluation

**Figure 4:**
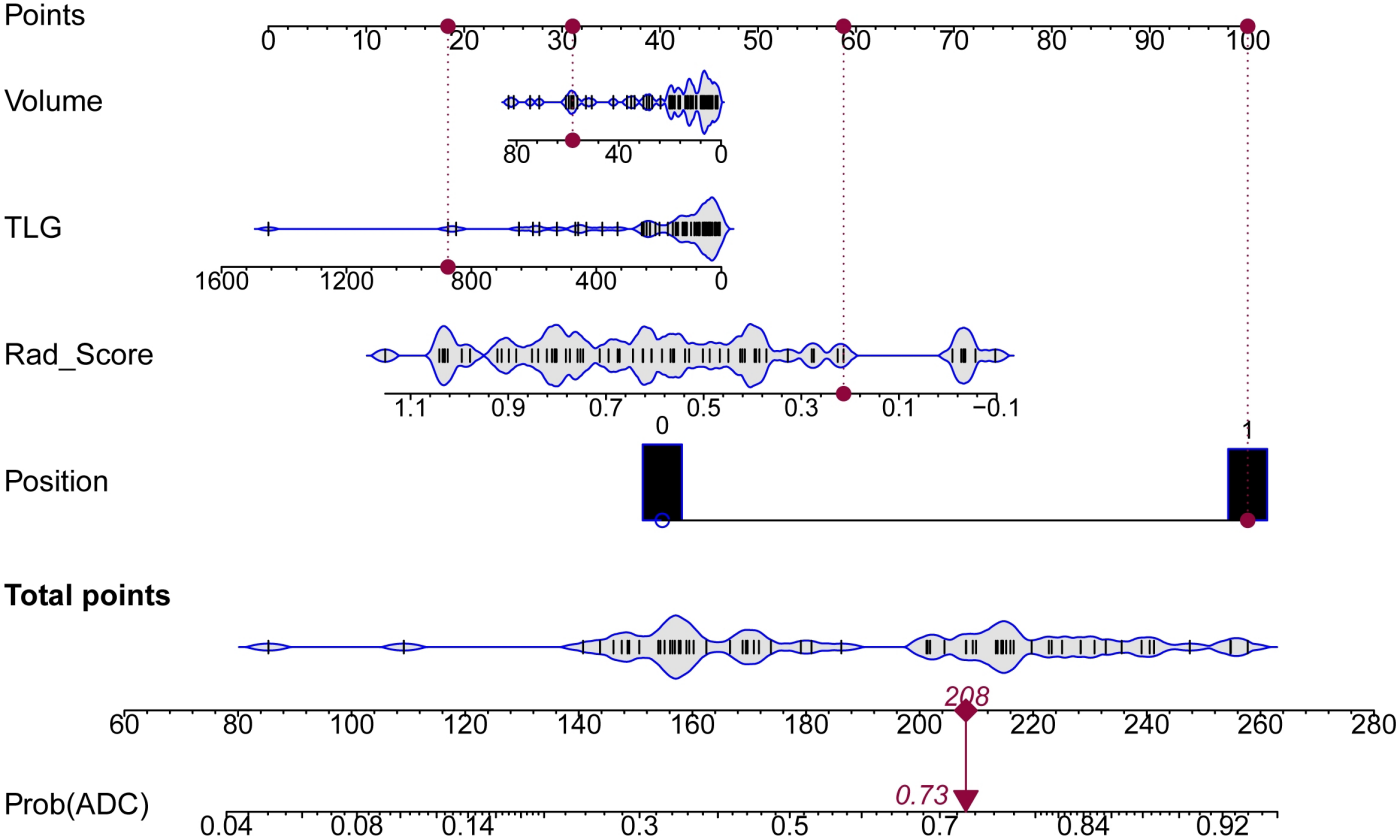
Nomogram for predicting the risk probability of ADC. Four variables were included in the nomogram model. For a given sample, each variable had a point, and the total point reflected the probability of ADC.

The detailed information of PET/MRI + CT + Clinical model (composite model) is displayed in **Formulas S1**, **S2** and **Figure S4**, including the formula of Rad_score (reflecting imaging information), the formula of Nomo_Score (representing imaging and clinical information), and Nomo_Score distribution in the ADC and SCC. Importantly, we constructed a nomogram to calculate individualized ADC probabilities for patients with lung cancer. As shown in **Figure 4**, Rad_Score was calculated from 15 imaging features, representing the key information of images of 3 modalities, while the volume, TLG and position were important clinical features. Thus, this nomogram combined tri-modality imaging information with clinical information. For a given patient, every variable corresponded to a point, and the total point corresponded to the probability of ADC.

**Figure 5:**
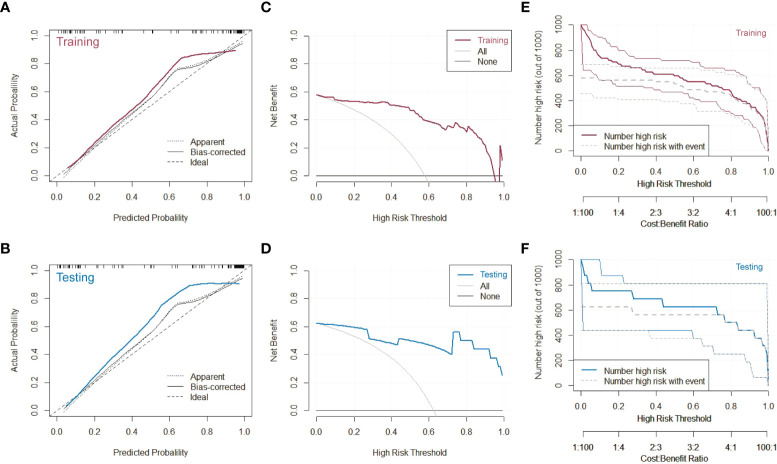
Characterizations of the PET/MRI + CT + Clinical prediction model. **(A)** Calibration curve, **(C)** decision curve and **(E)** clinical impact curve of the model on the training dataset. **(B)** Calibration curve, **(D)** decision curve and **(F)** clinical impact curve of the model on the testing dataset.

The calibration curves, decision curves and clinical impact curves were plotted to evaluate the discriminative performance of the composite model, as shown in **Figure 5**. Relevant characterizations could not be carried out in the internal testing set and external testing set due to the limited data. Thus, we combined them as the testing group. As shown in **Figures 5A**, the PET/MRI + CT + Clinical prediction model fitted well with the actual disease in the calibration curves to a certain extent, in both the training and testing sets. Most notably, the composite model could classify the ADC and SCC over a wide range. Furthermore, we created the decision curves **(** **Figures 5C** **)** and clinical impact curves **(** **Figures 5E** **)** in both the training and testing sets to explore the clinical benefit of the prediction model. The clinical net benefit was the difference between the benefits of intervention to true-positive patients and the costs of intervention to false-positive patients, which was useful for determining whether clinical decisions based on the prediction model would do better than harm. The decision curves were estimates of the standardized net benefit by the probability threshold used to categorize observations as “high risk”. Clinical impact curve analysis was also performed to evaluate the clinical applicability. For a wide range of the high-risk threshold, the PET/MRI + CT + Clinical prediction model had higher clinical net benefits in both the training and testing datasets. All these results verified that the composite model had superior predictive performance, achieving high clinical benefits and helping clinicians make clinical decisions.

## Discussion

We constructed nine different prediction models based on 80 cases and found that the PET/MRI + CT + Clinical model had the best predictive efficacy for lung adenocarcinoma and lung squamous cell carcinoma. Based on this model, we obtain the most relevant features, of which (mri-GLSZM-GLN) and (pet_shotnoise_GLCM_IDM) account for a large weight ratio. GLSZM quantifies gray-level zones in the image, which is inversely correlated with survival and helps identify the hypoxic or necrotic areas with poor prognosis. A gray-level zone is defined as the number of connected voxels sharing the same gray-level intensity. It measures the variability of gray-level intensity values in the image, with a lower value indicating more uniform intensity values (https://pyradiomics.readthedocs.io/en/latest/features.html). The results of our study indicated that ADC was more homogeneous than SCC, which could indirectly reflect the structure of the tumor itself. ADC was mainly composed of glandular structures (such as glandular duct and glandular cavity-like structures), while SCC was mainly composed of cornified beads, cancer nests and intercellular bridges. Besides, ADC had various growth modes, relatively few tumor cells per unit volume with abundant stroma, low water content, and relatively uniform distribution. In contrast, SCC had a tighter structure with more tumor cells per unit volume, a higher water content, and uneven distribution of various components, which made it easy to necrosis and cystic degeneration, leading to relatively uneven density. The results were consistent with those of Orlhac et al. (21), who found that scaly cell carcinoma had lower homogeneity and higher entropy by comparing the texture characteristics of ADC and SCC. Moreover, it was consistent with our previous findings (15) in that PET/MRI was used to create a prediction model for the pathological subtypes of ADC and lung SCC. It was also found that the GLSZM-GLN feature value accounted for the maximum weight ratio, indicating that ADC was more homogeneous than SCC. The GLCM of size Ng × Ng described the second-order joint probability function of an image region constrained by the mask. It was a measure of the local homogeneity of an image. IDM weights were the inverse of the contrast weights (decreasing exponentially from the diagonal i = j in the GLCM) (22). Therefore, in this study, the pet_shotnoise_GLCM_IDM weight ratio was large, indicating that ADC had higher homogeneity than SCC.

In our study, the most relevant parameters in the clinical model for the pathological classification of ADC and SCC were volume, TLG, and position, which was consistent with published findings. Koh et al. (23) retrospectively analyzed 269 cases of NSCLC preoperative PET-CT imaging and found significant differences in metabolic volume (MTV), TLG values, and GLUT1 expression between patients with lung ADC and those with SCC. Lu et al. (24) also discovered that the factors such as MTV, TLG, SUV histogram width, and texture characteristics were more valuable than traditional SUVmax value changes in predicting the tumors in clinical practice. This might be related to the heterogeneity of ADC and SCC, which varied in their growth rate and mode of infiltration into the surroundings, resulting in different morphologies and sizes.

18F-FDG PET can be used to determine the metabolic activity of lung tumors, but it is prone to false-negative or false-positive re the metabolic activity of lung tumors, but it is prone to false-positive and false-negative resultsesults. False-negative results could be seen in small tumors and bronchogenic carcinoma, while false-positive results are seen in cases of infection or inflammation. Reinfeld et al. (25) found that non-cancer cells in the tumor microenvironment were predominantly glucose-dependent macrophages, whereas tumor cells were predominantly glutamine-dependent, through the use of two different PET tracers. Therefore, the basis of PET tumor imaging might be the result of competition between tumor cells and macrophages. Due to the heterogeneity of ADC and SCC, differences were observed in TLG in different pathological types of lung cancer. Davide et al. (26) reported that SUVmean, SUVmax, and TLG were correlated significantly with disease-free survival. On the contrary, the bronchi below the segment (around the lung) of ADC and the bronchi above the segment (near the hilum) of SCC were satisfactory. Therefore, different pathological subtypes of lung cancer were considered associated with the location of the tumor.

In recent years, many researchers have established image prediction models for the pathological classification of lung cancer. Kirienko et al. (27) established PET radiomics models and CT radiomics models for predicting ADC and SCC. The AUC value of the PET radiomics model was 0.90 ± 0.10 and 0.80 ± 0.04 in the training group and testing group, respectively; while the AUC value of the CT radiomics model was 0.81 ± 0.02 and 0.69 ± 0.04 in the training group and testing group, respectively. It confirmed that the imaging models based on PET or CT could predict lung cancer subtypes well and distinguish primary and metastatic lung lesions. Caiyue et al. (28) found that the machine learning–integrated ^18^F-FDG PET/CT radiomics model based on the clinical characteristics of 315 patients with NSCLC could efficiently predict the pathological set of SCC and ADC, with an AUC of 0.932 (95% CI: 0.900–0.964) and 0.901 (95% CI: 0.840–0.957) in the training set and testing set, respectively. In our previous study (15), 61 patients with ADC or SCC were divided into a training group and a testing group at the ratio of 7:3, and the features selected from preoperative PET/MRI images were applied to create a prediction model. It was found that the AUC value for classifying ADC and SCC was 0.886 (95% CI: 0.787–0.985) and 0.847 (95% CI: 0.648–1.000) in the training and testing groups, respectively. A common feature compared with previous studies was that all image-based prediction models could predict the pathological subtypes of ADC or SCC well. The difference in this study was that we established nine different prediction models, and every prediction model had a high diagnostic value for the pathological types of ADC and SCC, especially the PET/MRI + CT + Clinical model. The AUC value of this composite model in the training group, internal testing group and external testing group was 0.965 (95% CI: 0.920–1.0), 0.933 (95% CI: 0.746–1.000) and 0.867 (95% CI: 0.593–1.000), respectively. Using DeLong’s test, it was found that the predictive performance of PET/MRI + CT + Clinical model was superior to that of other prediction models in the training set, except PET/MRI + CT model. Based on the best model, a nomogram was constructed to visualize the probability of ADC. Moreover, the calibration curves, decision curves, and clinical impact curves verified that the PET/MRI + CT + Clinical prediction model owned great discriminative performance and high clinical net benefit. This might be attributed to two reasons: 1) The model could distinguish subtle structures by integrating the multi-parameter multifunctional imaging of MRI, the metabolic characteristics of PET, and high-resolution CT; 2) The model combined tri-modal imaging features and clinical features could accurately display the shape of the lesion and obtain the pathological and physiological information of the tumor so as to achieve efficient prediction of the pathological type of lung cancer.

This study had several limitations. First, this was a single-center retrospective study with a small sample size. Hence, designing new multicenter cooperative prospective studies was still necessary. Second, the sample selection had a bias. Some patients with NSCLC, especially those with ADC, were excluded from radiomics analysis due to their weak ^18^F-FDG uptake or small tumor volume so as to ensure the quality of image and texture data. With the increasing use of imaging screening for lung cancer, small lesions were more likely to be detected in the early stage. Furthermore, ROI manual segmentation is time-consuming and vulnerable to the inconsistency of different readers. Although automatic and semi-automatic segmentations have been used to increase objectivity and minimize time cost, no specification is available to guide or assess the efficiency of segmentation.

## Conclusions

In conclusion, based on the PET/MRI + CT + Clinical prediction model, ADC or SCC could be well differentiated preoperatively, since it was non-seminal and repeatable and had clinical practicability. The model integrated multi-modal imaging features and clinical features showed great potential in predicting the pathological subtype, thus further helping clinicians make decisions.

## Data availability statement

The original contributions presented in the study are included in the article/**Supplementary Material**. Further inquiries can be directed to the corresponding authors.

## Ethics statement

The studies involving human participants were reviewed and approved by Scientific Research Medical Ethics, No. 2021-008. The patients/participants provided their written informed consent to participate in this study.

## Author contributions

Guarantor of integrity of the entire study: ZD and FS. Study concepts: XT and JW. Study design: XT and JW. Literature research: XT and JW. Data acquisition: XT, JL, and CY. Statistical analysis: JW and FS. Manuscript preparation: XT and JW. Manuscript editing: XT. Manuscript review: ZD and FS. All authors contributed to the article and approved the submitted version.

## Funding

This study was supported by National Natural Science Foundation of China (81871337); Natural Science Foundation of Zhejiang Province (LY16H180007).

## Conflict of interest

JW and FS are employees of Shanghai United Imaging Intelligence Co., Ltd.

The remaining authors declare that the research was conducted in the absence of any commercial or financial relationships that could be construed as a potential conflict of interest.

## Publisher’s note

All claims expressed in this article are solely those of the authors and do not necessarily represent those of their affiliated organizations, or those of the publisher, the editors and the reviewers. Any product that may be evaluated in this article, or claim that may be made by its manufacturer, is not guaranteed or endorsed by the publisher.

## Supplementary material

Supplementary Figure 1 Selected features from three imaging modalities and clinical data. **(A)** PET image; **(B)** CT image; **(C)** MRI image; and **(D)** clinical features.

Supplementary Figure 2 ROC curves of different models in the training set, internal testing set, and external testing set. **(A)** PET model; **(B)** CT model; **(C)** MRI model; **(D)** Clinical model; **(E)** PET + CT model; **(F)** PET/MRI model; **(G)** PET + Clinical model; **(H)** PET/MR + CT model; and **(I)** PET/MR + CT + Clinical model.

Supplementary Figure 3 Comparison of AUC values of different models in the training set using DeLong’s test. *P* < 0.05 indicated a statistically significant difference. Rad_Score = 0.1551 * mri_log_gldm_log.sigma.1.5.mm.3d.dependencenonuniformitynormalized + 0.1332 * pet_shotnoise_glcm_idmn + 0.0920 * ct_wavelet_firstorder_wavelet.hll.skewness + 0.0278 * pet_normalize_glrlm_runlengthnonuniformity + 0.0108 * ct_wavelet_ngtdm_wavelet.hhh.contrast - 0.0042 * ct_log_glszm_log.sigma.0.5.mm.3d.largearealowgraylevelemphasis - 0.0056 * pet_normalize_ngtdm_busyness - 0.0397 * mri_normalize_gldm_dependencevariance - 0.0456 * ct_wavelet_gldm_wavelet.hll.largedependencehighgraylevelemphasis - 0.0477 * mri_wavelet_glcm_wavelet.llh.imc2 - 0.0514 * pet_wavelet_glcm_wavelet.hhl.jointentropy - 0.0604 * ct_wavelet_gldm_wavelet.hlh.largedependencehighgraylevelemphasis - 0.0643 * pet_wavelet_firstorder_wavelet.llh.kurtosis - 0.0837 * mri_specklenoise_ngtdm_complexity - 0.1476 * mri_wavelet_glszm_wavelet.lhh.graylevelnonuniformity + 0.5833

Supplementary Figure 4 Nomo_Score of ADC and SCC in the training set **(A)** and internal testing set **(B).** Nomo_Score was generated from PET/MRI + CT + Clinical prediction model, combing imaging information and clinical information. Label 0 represented SCC, and Label 1 represented ADC. The results demonstrated that the two diseases could be differentiated well using the Nomo_Score.
