# Supplementary figures and images for: The value of combined PET/MRI, CT and clinical metabolic parameters in differentiating lung adenocarcinoma from squamous cell carcinoma
Source: Front Oncol. 2022 Aug 23;12:991102. doi: 10.3389/fonc.2022.991102 (PMC9445186; doi:10.3389/fonc.2022.991102)

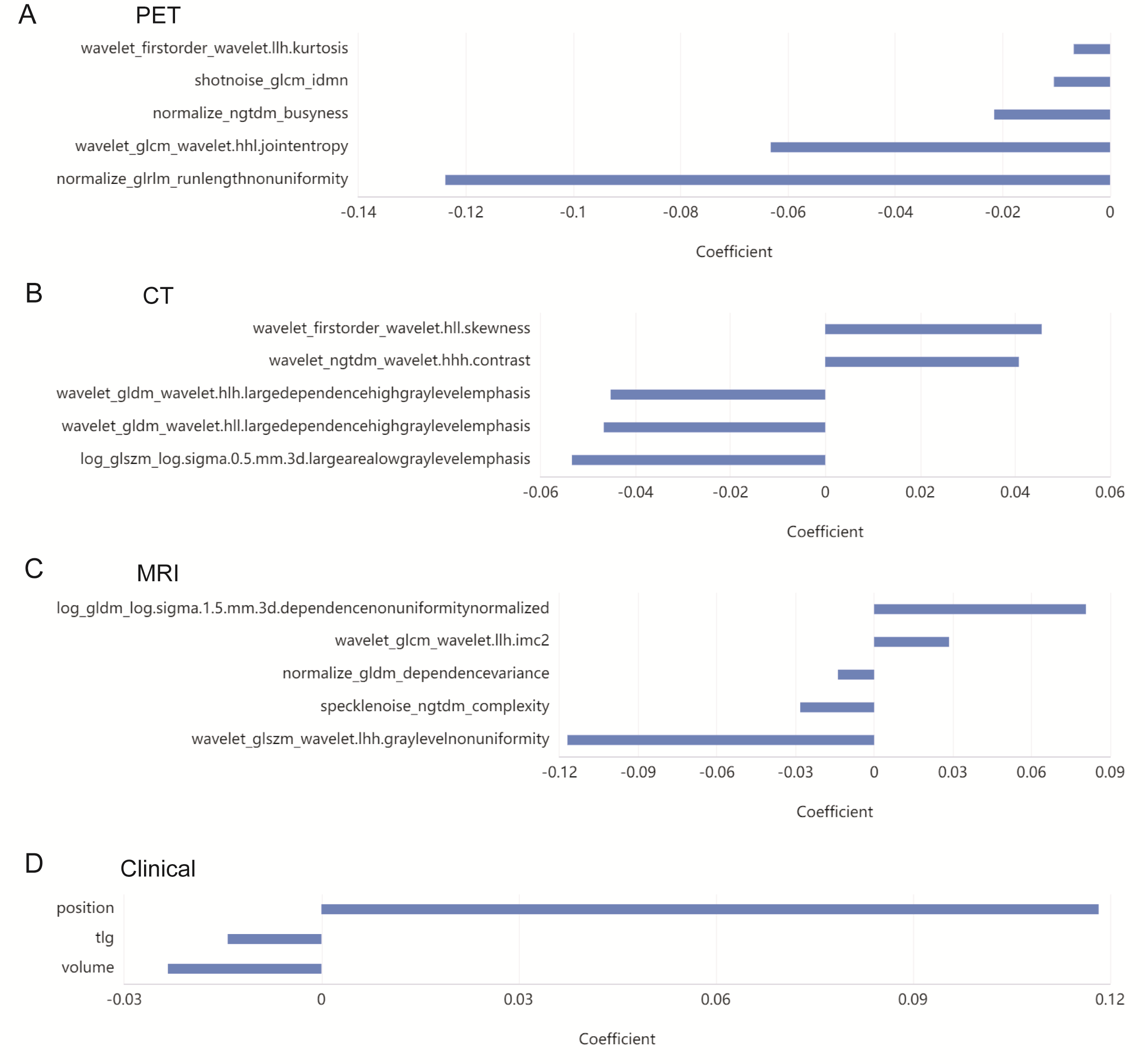

Supplement: Supplementary Figure 1 — Selected features from three imaging modalities and clinical data. (A) PET image; (B) CT image; (C) MRI image; and (D) clinical features. [file Image_1.png]

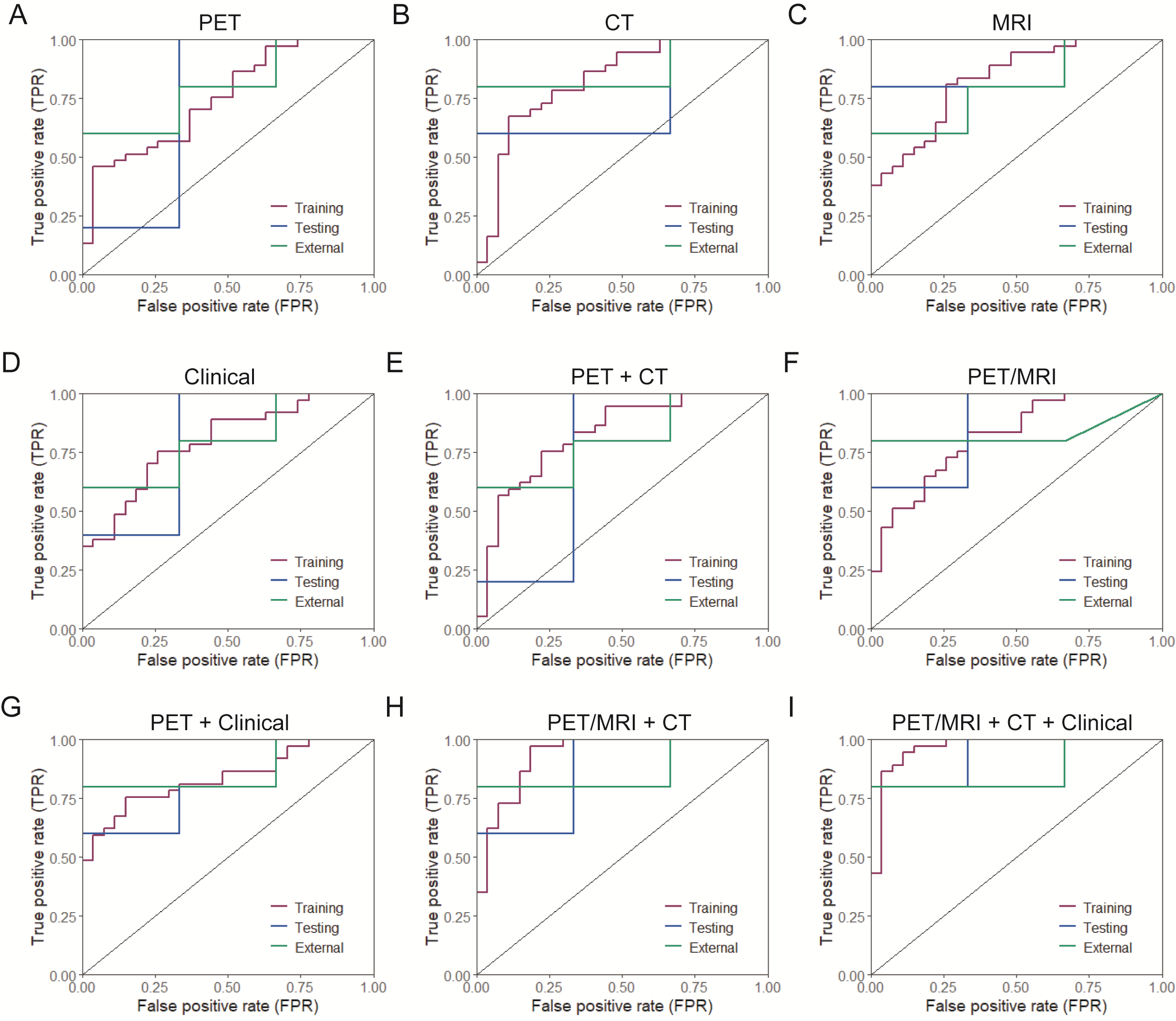

Supplement: Supplementary Figure 2 — ROC curves of different models in the training set, internal testing set, and external testing set. (A) PET model; (B) CT model; (C) MRI model; (D) Clinical model; (E) PET + CT model; (F) PET/MRI model; (G) PET + Clinical model; (H) PET/MR + CT model; and (I) PET/MR + CT + Clinical model. [file Image_2.png]

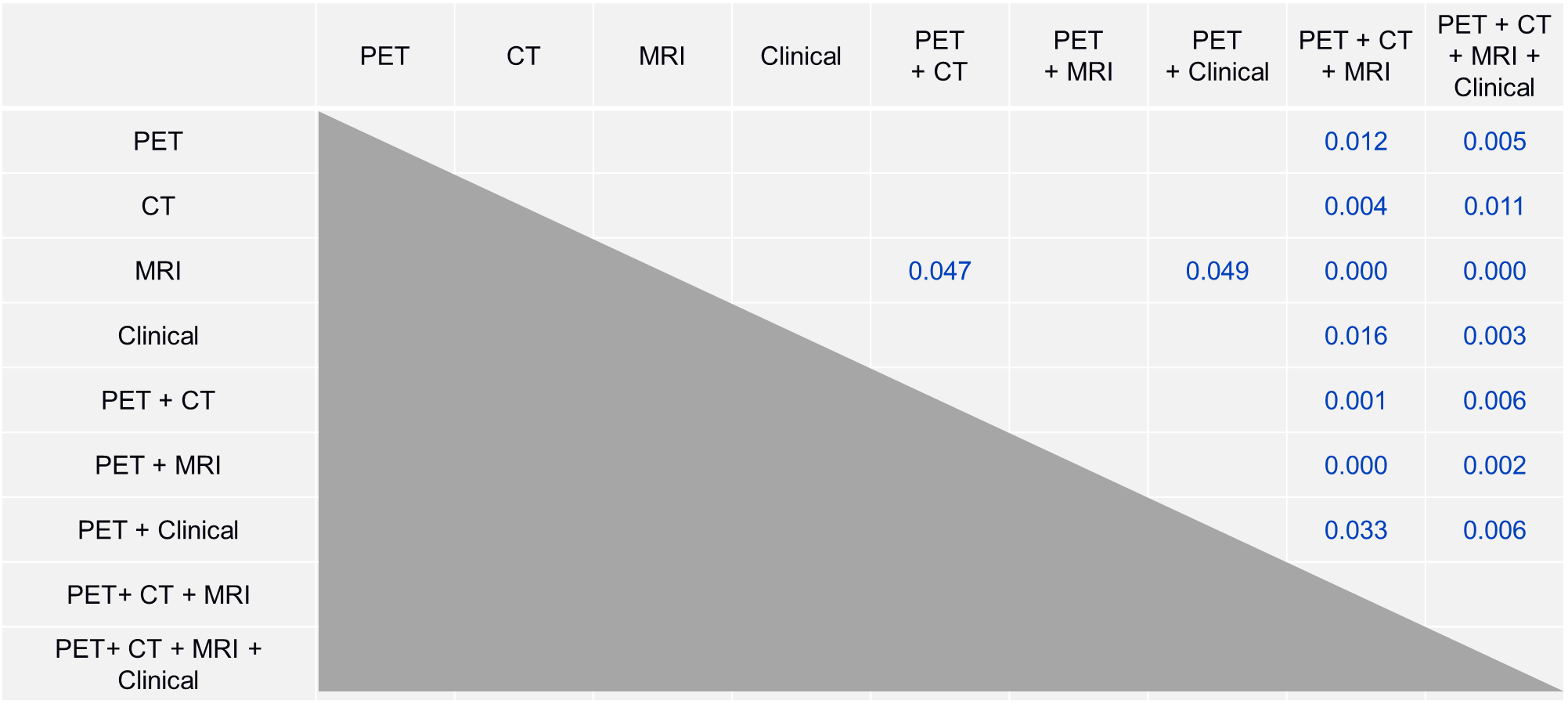

Supplement: Supplementary Figure 3 — Comparison of AUC values of different models in the training set using DeLong’s test. P < 0.05 indicated a statistically significant difference. Rad_Score = 0.1551 * mri_log_gldm_log.sigma.1.5.mm.3d.dependencenonuniformitynormalized + 0.1332 * pet_shotnoise_glcm_idmn + 0.0920 * ct_wavelet_firstorder_wavelet.hll.skewness + 0.0278 * pet_normalize_glrlm_runlengthnonuniformity + 0.0108 * ct_wavelet_ngtdm_wavelet.hhh.contrast - 0.0042 * ct_log_glszm_log.sigma.0.5.mm.3d.largearealowgraylevelemphasis - 0.0056 * pet_normalize_ngtdm_busyness - 0.0397 * mri_normalize_gldm_dependencevariance - 0.0456 * ct_wavelet_gldm_wavelet.hll.largedependencehighgraylevelemphasis - 0.0477 * mri_wavelet_glcm_wavelet.llh.imc2 - 0.0514 * pet_wavelet_glcm_wavelet.hhl.jointentropy - 0.0604 * ct_wavelet_gldm_wavelet.hlh.largedependencehighgraylevelemphasis - 0.0643 * pet_wavelet_firstorder_wavelet.llh.kurtosis - 0.0837 * mri_specklenoise_ngtdm_complexity - 0.1476 * mri_wavelet_glszm_wavelet.lhh.graylevelnonuniformity + 0.5833 [file Image_3.png]

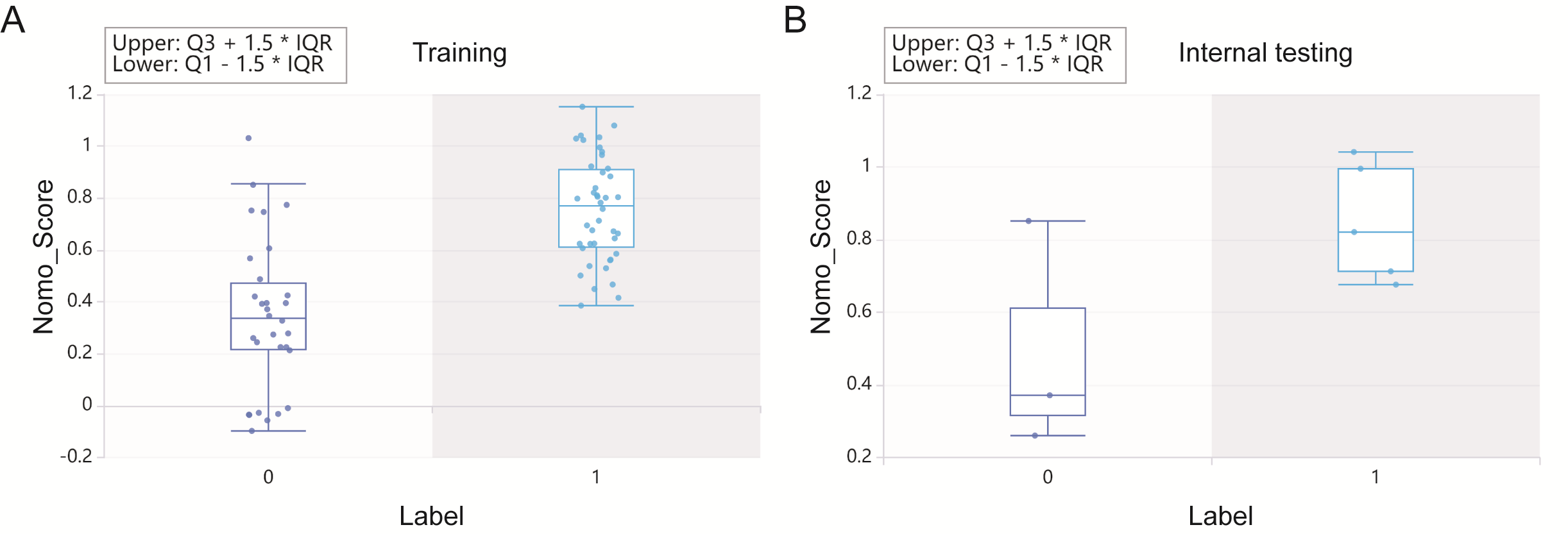

Supplement: Supplementary Figure 4 — Nomo_Score of ADC and SCC in the training set (A) and internal testing set (B). Nomo_Score was generated from PET/MRI + CT + Clinical prediction model, combing imaging information and clinical information. Label 0 represented SCC, and Label 1 represented ADC. The results demonstrated that the two diseases could be differentiated well using the Nomo_Score. [file Image_4.png]
